# Mechanical network equivalence between the katydid and mammalian inner ears

**DOI:** 10.1371/journal.pcbi.1012641

**Published:** 2024-12-13

**Authors:** Emine Celiker, Charlie Woodrow, Òscar Guadayol, Leonidas-Romanos Davranoglou, Christian M. Schlepütz, Beth Mortimer, Graham K. Taylor, Stuart Humphries, Fernando Montealegre-Z

**Affiliations:** 1 School of Engineering, University of Leicester, Leicester, United Kingdom; 2 Department of Ecology and Genetics, Uppsala University, Uppsala, Sweden; 3 Mediterranean Institute for Advanced Studies, IMEDEA (UIB-CSIC), Mallorca, Spain; 4 Oxford University Museum of Natural History, University of Oxford, Oxford, United Kingdom; 5 Swiss Light Source, Paul Scherrer Institute, Villigen PSI, Switzerland; 6 Department of Biology, University of Oxford, Oxford, United Kingdom; 7 School of Life and Environmental Sciences, University of Lincoln, Lincoln, United Kingdom; Friedrich Schiller University Jena, GERMANY

## Abstract

Mammalian hearing operates on three basic steps: 1) sound capturing, 2) impedance conversion, and 3) frequency analysis. While these canonical steps are vital for acoustic communication and survival in mammals, they are not unique to them. An equivalent mechanism has been described for katydids (Insecta), and it is unique to this group among invertebrates. The katydid inner ear resembles an uncoiled cochlea, and has a length less than 1 mm. Their inner ears contain the *crista acustica*, which holds tonotopically arranged sensory cells for frequency mapping via travelling waves. The *crista acustica* is located on a curved triangular surface formed by the dorsal wall of the ear canal. While empirical recordings show tonotopic vibrations in the katydid inner ear for frequency analysis, the biophysical mechanism leading to tonotopy remains elusive due to the small size and complexity of the hearing organ. In this study, robust numerical simulations are developed for an *in silico* investigation of this process. Simulations are based on the precise katydid inner ear geometry obtained by synchrotron-based micro-computed tomography, and empirically determined inner ear fluid properties for an accurate representation of the underlying mechanism. We demonstrate that the triangular structure below the hearing organ drives the tonotopy and travelling waves in the inner ear, and thus has an equivalent role to the mammalian basilar membrane. This reveals a stronger analogy between the inner ear basic mechanical networks of two organisms with ancient evolutionary differences and independent phylogenetic histories.

## Author summary

Katydids (Insecta) have a unique hearing system among invertebrates because, similar to mammals, they exhibit outer, middle, and inner ear components. The katydid inner ear resembles an uncoiled mammalian cochlea. Although much smaller in size (∼700 *μ*m), the katydid inner ear possesses a tonotopic frequency-discriminating sensory epithelium like the mammalian cochlea. Yet, the mechanical equivalence between the workings of the two inner ears has not been investigated experimentally, due to the minuscule dimension and tightly integrated inner ear components of the katydid ear. In this study, comprehensive numerical simulations are developed using accurate 3D ear models to investigate such an equivalence. The results reveal that similar to the mammalian basilar membrane, the triangular structure holding the katydid hearing organ drives the movements of the mechanosensors, so that the minuscule katydid inner ear functions with a similar biophysical mechanism as the mammalian cochlea. This provides a unique opportunity to infer processes difficult to measure in the mammalian cochlea through an analogous model auditory system.

## Introduction

**Fig 1:**
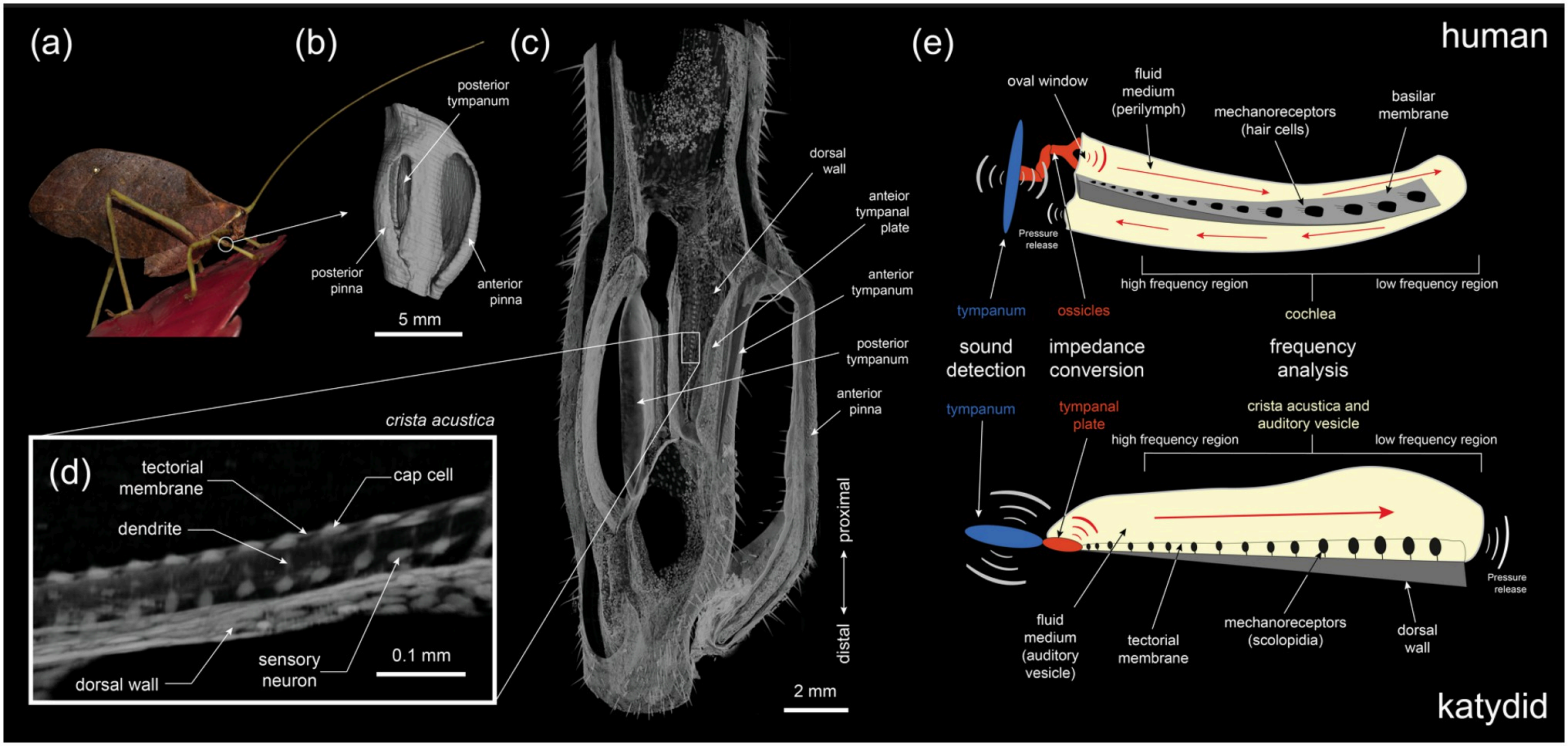
*Mimetica* sp. ear. (a) An actual image of the bush-cricket *Mimetica* sp.; (b) The pinna covering the tympanal membranes of *Mimetica* sp., located at the femoro-tibial joint, obtained from 3D-volume renders of synchrotron micro-CT scans; (c) The hearing organ (*crista acustica*) located on the dorsal wall tapering in width, obtained from 3D-volume renders of synchrotron micro-CT scans; (d) A zoomed-in image of the *crista acustica* components, obtained from 3D-volume renders of synchrotron micro-CT scans; (e) A drawing of the human and katydid middle and inner ears for comparison.

The spatial arrangement of processing sounds of different frequencies in the auditory system, or tonotopy, was first discovered in the inner ear of mammals by Georg von Bèkèsy [1]. In the mammalian inner ear (the cochlea), mechanosensory receptors are located on the basilar membrane which tapers in width and gradually increases in thickness and stiffness (Fig 1e), leading to stiffness and mass gradients along the structure [2]. These structural gradients provide the main constituents for a tonotopic arrangement through systematically changing resonance properties (see [2] and references therein). A consequence of this gradient is the generation of a basilar membrane travelling wave upon sound-induced pressure changes in the fluid-filled cochlea [3]. The membrane then acts as a biological Fourier transform, performing a frequency analysis of the travelling wave through displacement amplitude maxima, or resonance, response to the frequency information [1, 2]. This displacement leads to the cochlear hair cells (or auditory sensory cells) lying along the membrane to receive mechanical input at specific frequencies. However, the precise role of cochlear hair cells and the cochlear fluid are challenging to identify non-invasively. [4–7].

Analogous tonotopic processes have been observed directly in the auditory systems of birds [8], and indirectly via the timing of responses of auditory-nerve fibres in the auditory organs of some reptiles and frogs [9, 10]. The katydid (or bushcricket) ear (see Fig 1) also has a tonotopic travelling wave [11–15] but the tightly integrated inner ear components and limitations of experimental techniques make it especially difficult to understand the biophysisical mechanisms underpinning the tonotopy. While it is not known to what extent the working of the katydid inner ear is equivalent to that of the mammalian cochlea, a mechanical equivalence between the two systems would certainly allow for a deeper understanding of both the systems, and hence merits further investigation.

Among insects, katydid ears are unique as they have evolved the same three basic hearing stages observed in mammals: 1) sound capturing, 2) impedance conversion, and 3) frequency analysis, facilitated by outer, middle and inner ear components, respectively [15, 16]. Katydids have an ultrasound sensitive hearing system, and detect sound through paired tympana located below the femoro-tibial joint of their prothoracic legs, which flank the inner ear (Fig 1(a)–1(c)). The sound waves reach the exposed external side of their tympana directly. A bifurcated tracheal tube is connected to, and delivers sound to the internal side of both tympana [16]. Sound, therefore, reaches the external side of the tympanic membranes but also the internal side through the trachea [17]. The katydid inner ear, the auditory vesicle (AV), is located on the flattened, gradually narrowing dorsal wall (DW) of the anterior branch (Fig 1(c)). The DW has a triangular shape increasing in width and decreasing in thickness [20], from the distal end to the proximal end (see Fig 1), leading to mass and stiffness gradients along its structure. The AV is a liquid-filled space that has an intersection with the tympanic membranes. At the region of intersection, the tympana of various katydid species host a hardened cuticular region called tympanal plates (TPs), which act as their middle ear [15, 16, 20]. More precisely, TPs facilitate the impedance conversion of the sound vibrations from the air-filled medium of the trachea to the liquid-filled medium of the AV [15]. These vibrations in turn activate the *crista acustica* (CA) which lies immersed in the AV fluid, on top of the DW. The CA is comprised of scolopidial units, where each scolopidium possesses a single dendrite, with a mechanosensory cilium at its distal end embedded in a cap cell (Fig 1(d)) [21]. The scolopidia are longitudinally coupled to two membranes, where the DW forms the lower border, and the cap cells are embedded in a membrane called the tectorial membrane that covers the whole of the CA. The tectorial membrane is supported by two ‘bands’ [18, 19], which, according to Olson and Nowotny [13] could be important for the function of a longitudinal coupled resonator of the sensory cells. The katydid inner ear thus resembles an uncoiled mammalian cochlea, and possesses a spatial organisation that allows for frequency dependent displacements activated by a travelling wave along the hearing organ, akin to the mammalian ear (Fig 1(e)). The characterisation of the travelling wave in the inner ear is based on three main response characteristics of the mammalian basilar membrane [1, 2]:

1. The movement of the vibrations along the hearing organ shows increasing phase lag as a function of stimulus frequency. Hence, in the frequency domain, this leads to a phase accumulation exceeding the high frequency phase-lag expected for simple resonators at the peak frequency.
2. Around the point of maximum displacement of the hearing organ, the leading slope of the envelope (the smooth curve outlining the extremes of the displacement oscillations) is steeper than the trailing slope, so that the displacement magnitudes have an asymmetric envelope around that point.
3. The travelling wave results from the mechanical characteristics of the propagating medium such as stiffness and mass gradients, so that the increase in amplitude is passive amplification.

The travelling wave and frequency dependent displacement of the katydid hearing organ was recorded directly for the first time by Udayashankar et al. through *in vivo* measurements, in the inner ear of the species *Mecopoda elongata* [22]. To access the hearing organ, the dorsal cuticle and the inner ear fluid was removed. The CA surface was then stimulated directly and the vibrations were monitored using a Laser Doppler Vibrometer (LDV). Montealegre et al. also reported the emergence of a travelling wave and mechanical tonotopy in the ear of the katydid *Copiphora gorgonensis* [15] and of other species with transparent cuticles [12] non-invasively. For this, the LDV recordings of the hearing organ vibrations were obtained through the transparent dorsal cuticle of these species. Recently, inner ear vibrations of *M. elongata* were recorded by optical coherence tomography (OCT) vibrometry non-invasively [11], showing a tuned periodic deformation of the CA and the DW. Further, Celiker et al. carried out a three-dimensional (3D) numerical simulation of tonotopy within the katydid inner ear [20] and numerically demonstrated that the stiffness and mass gradients along the hearing organ allowed for tonotopy.

Following the observation of tonotopy in the katydid inner ear, much time has also been devoted to investigating the underlying mechanism behind it. For instance, by investigating the morphology and physiology of the auditory receptor organs of three tettigoniid species, Kalmring et al. [23] suggested the DW acted as the source of tonotopy. Hummel et al. [21], however, after obtaining detailed anatomical measurements of the CA components proposed that tonotopy is induced from the combined mass of the cap cells and stiffness gradients of rootlets within the dendrites. Despite detailed correlative morphology it is impossible to experimentally manipulate the auditory system to systematically test the relative contribution of the different components. In this study, to understand the biomechanism underlying the katydid inner ear and go beyond experimental limitations, we developed comprehensive and robust 3-dimensional (3D) finite element models of the katydid inner ear processes. The geometry which was used as the solution domain of the mathematical models was the precise geometry of the species *Mimetica* sp., obtained through synchrotron-based Micro-Computed Tomography (*μ*–CT) Scanning and 3D reconstruction. To our knowledge, this is the first comprehensive numerical simulation of the katydid inner ear that incorporates the precise geometry of the ear. The model also incorporated AV fluid properties obtained *in situ* via multiple particle tracking microrheology. We validated the models through comparisons with experimental data, and then used numerical simulations to go beyond the experimental limitations and test the hypothesis that the driving force behind the observed tonotopy along the hearing organ is the displacement of the DW.

## Materials and methods

### Inner ear geometry

The ear of *Mimetica* sp. was scanned using the TOMCAT X02DA beamline at the Swiss Light Source. The ear was mounted in an inverted micro pipette tip filled with 100% ethanol following an ascending dehydration series in 20% steps. Super glue was used to hold the pipette tip to the mounting stage. Before scanning, the sample was de-gassed in a vacuum oven at 35°C to remove bubbles within the sample. The pipette tip was then mounted in the beamline and scanned at an isotropic voxel spacing of 1.625 *μ*m. The tomographic data was collected using the parameters listed in Fig A in S1 Text. To process the tomographic data, the image slices were also first batch converted in ImageJ [24], to reduce the file size (to 75%). The file size was reduced by cropping along the *x* − *y* axes to only include the region of interest. The new down-sampled dataset was then imported into Amira-Aviso 6.7 (Thermo Fisher Scientific, Waltham, Massachusetts, USA) for segmentation. For segmentation of large structures (AV, DW, TP, tectorial membrane), slices were semi-automatically highlighted every 5 slices followed by interpolation. For small structures (dendrites, cap cells), each individual slice was segmented to ensure the best possible accuracy and resolution in the geometry. All segmented structures were then exported as Standard Tessellation Language (STL) files for modelling.

### Auditory vesicle fluid properties

Density of the AV fluid from eight specimens of the katydid *Copiphora gorgonensis* was measured using a volumetric method. The whole content of the AV fluid was drawn using a pneumatic microinjection system (PV830 Picopump, WPI) into borosilicate glass capillaries of 0.5 mm inner diameter previously pulled with a P-97 micropipette puller (Sutter Inc.). Capillaries were weighted at room temperature before and after fluid withdrawal on a microbalance (Sartorius Cubis MSA6.6S-000-DM) with a scale interval of 0.001mg to assess the weight of the withdrawn fluid. Volume of fluid was estimated from images taken with a Alicona InfiniteFocus 3D imaging system under controlled temperature. Retrieved volume ranged from 4 to 80 nL. Density was estimated as the ratio between weight and volume. For comparison and control, a similar procedure was used to measure the density of ultrapure water (Fig F(a) in S1 Text).

To characterize the rheological behaviour of AV fluid in katydids, we used *in situ* multiple particle tracking microrheology (Fig F(b) in S1 Text). Briefly, specimens of *C. gorgonensis* were immobilised on a glass slide with putty-like pressure-sensitive adhesive for the duration of the experiment. Then, a volume of less than 1 pL of a highly concentrated aqueous suspension of 1.02 *μ*m fluorescent microspheres (Fluoresbrite YG Carboxylate Microspheres, Polysciences Inc.) was injected into the AV using a pneumatic microinjection system (PV830 Picopump, WPI). Specimens were then placed on an inverted epifluorescence microscope (Zeiss AxioVert A1 Fl), which is equipped with an LED source (CoolLED pE-300 white) and a temperature control stage, and mounted on an antivibration table to eliminate environmental noise. Fluorescent microspheres were easily observed through the semi-transparent cuticle of the insects. Videos were then recorded at 400X magnification with a long-distance objective (ZeissLD Plan-Neofluor 40x/0.6NA Corr) with an ultra high speed camera (Photron FASTCAM MINI UX100) operating at 50Hz. Microspheres were tracked as they underwent Brownian motion using in-house Matlab code, [25]. Using this procedure we were able to characterize the rheological behaviour at frequencies between ∼ 10^−2^ and 25 Hz.

### Mathematical model

The system of equations used for representing the workings of the *Mimetica* sp. inner ear is given in detail in Section B of S1 Text. Below we give a worded summary of the system.

The numerical solutions were considered in both the frequency domain and the time domain. The katydid inner ear was excited by the vibrations of the tympanal plates with a prescribed acceleration condition of magnitude 10*ω*^2^ *μ*m/s^2^, where *ω* = 2*πf* is the angular frequency of the incoming vibrations and *f* is the frequency. In the frequency domain, the frequency was taken in the interval 10–80 kHz with a resolution of 10 kHz. The AV fluid was assumed to be a Newtonian fluid of density and viscosity similar to water. The pressure in the fluid was represented by the solution to the linearized Navier-Stokes equations, and thus viscous losses were accounted for [26].

**Fig 2:**
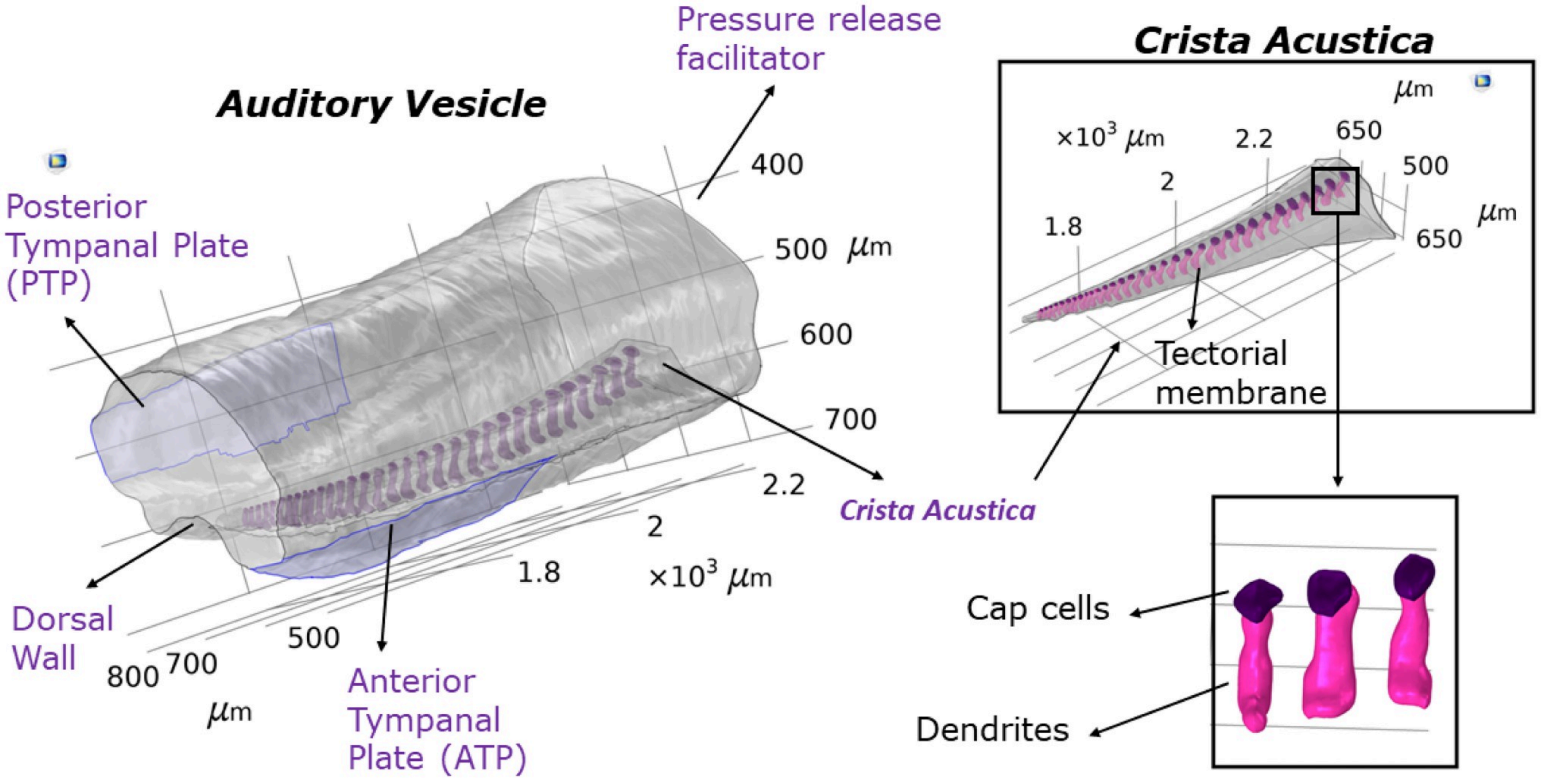
Model solution domain. The image of the *Mimetica* sp. inner ear geometry forming the solution domain of the mathematical model, as obtained by synchrotron-based *μ* − CT scans and 3D-reconstruction.

The structural components included in the model were the AV wall, the dorsal wall, and the CA components such as dendrites, cap cells and the tectorial membrane covering these components (Fig 2). All structural components were assumed to be linear elastic. The AV wall and the tectorial membrane were represented using a shell formulation, [27], with a thickness determined through *μ*− CT measurements (Table 1). The displacements of cap cells and dendrites were represented by the solution to the elastic Helmholtz equation [28] in the frequency domain, and the elastic wave equation in the time domain. The cap cells and dendrites were connected with a continuity condition, and then coupled with the tectorial membrane and the dorsal wall. Using the Arbitrary Lagrangian-Eulerian formulation, the structural components were also fully coupled with the fluid system.

**Table 1:** The Young’s modulus and thickness of components used in the mathematical model.

Parameter	Young’s Modulus (GPa)	Thickness (*μ*m)
Dorsal wall	0.003	4.10–4.84 (proximal to distal)
Acoustic vesicle wall	15	15
Cap cell	0.01	-
Dendrite	0.02	-
Tectorial membrane	0.05	3.08–3.47 (proximal to distal)
Tympanal plate	1	10
Pressure release facilitator	0.025	0.1

Table 1 lists the material properties incorporated into the mathematical models for the structural components, which were assumed to be isotropic and homogeneous. The listed Young’s moduli were selected using parametric sweeps and a comparison of the numerical results with the experimental data (Section D in S1 Text). The AV wall was assigned a free boundary condition [29], and hence was allowed to move based on its material properties and incoming forces. The only fixed constraints were placed along the edges of the AV faces. The AV face acting as a pressure release facilitator (Fig 2) was assigned different material properties to represent its function (see [15]). The thickness of the pressure release facilitator was also determined using parametric sweeps in the range 0.01-5 *μ*m, where the value used gave the best qualitative match of the numerical results with the experimental data. More precisely, we checked for a coupled tonotopic displacement of the CA and the DW, [11].

Details related to the numerical simulations are given in Section C of S1 Text.

## Results

### Numerical simulation of tonotopy in the katydid inner ear

**Fig 3:**
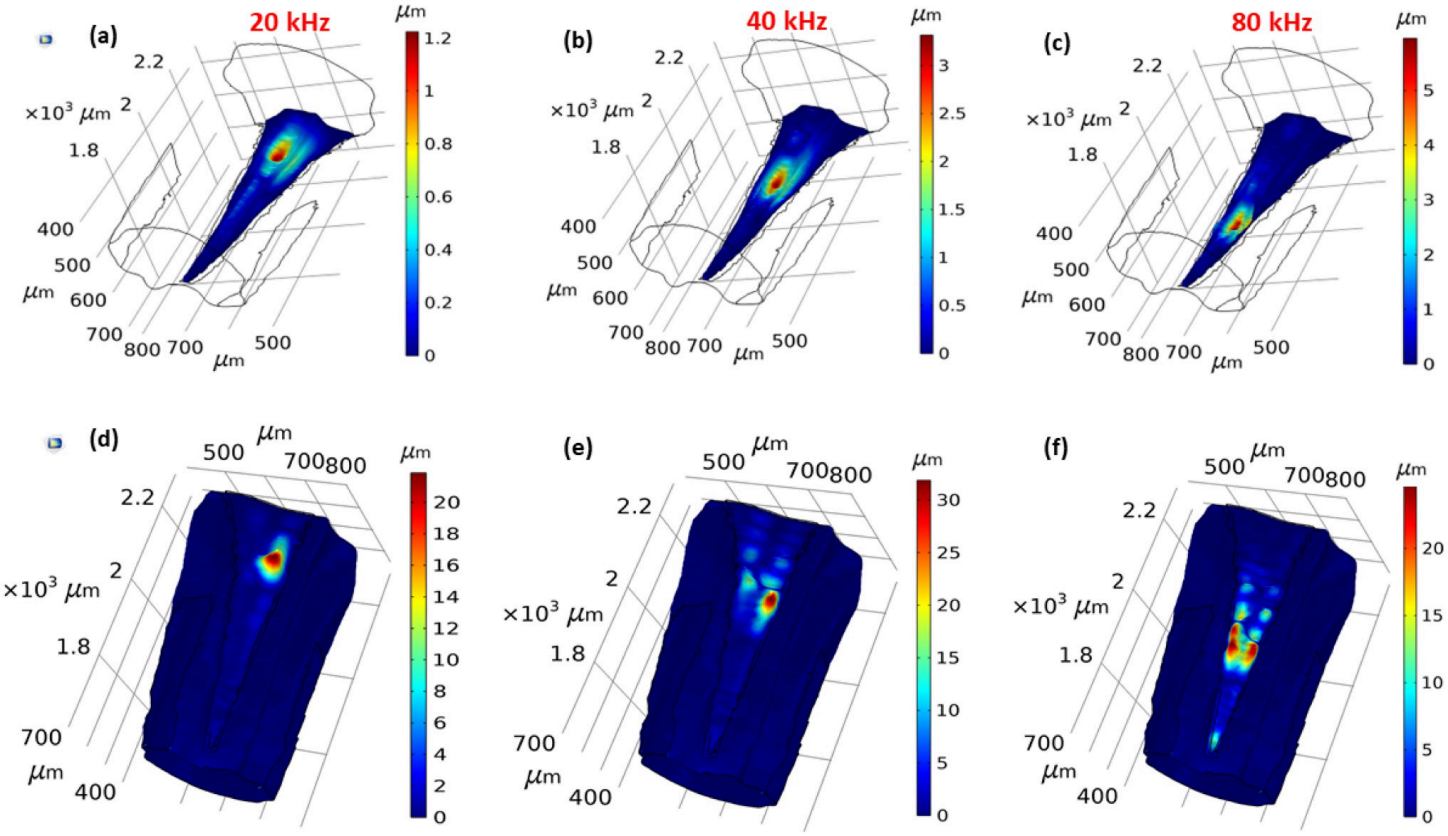
Tonotopy in the *Mimetica* sp. inner ear. The displacement magnitude along the *crista acustica* at (a) 20 kHz, (b) 40 kHz, and (c) 80 kHz. The displacement magnitude along the dorsal wall at (d) 20 kHz, (e) 40 kHz, and (f) 80 kHz.

The numerical results obtained in the frequency domain gave the displacement response of the DW and the CA to the vibrations entering the inner ear. We examined the presence of tonotopy in the *Mimetica* sp. inner ear by checking for an increase of the displacement maxima location from the proximal end along the dorsoventral axis, (see Fig 1) as the frequency increased from 10 kHz to 80 kHz. From the 3D results of the maximum displacement location at frequencies 20, 40 and 80 kHz on the CA (Fig 3(a)–3(c)) and the DW (Fig 3(d)–3(f)), displacement maxima of both the DW and the CA moved towards the distal end with the increase of frequency. The displacement of the cap cells and dendrites at 20, 40 and 80 kHz are demonstrated in Fig C in S1 Text.

**Fig 4:**
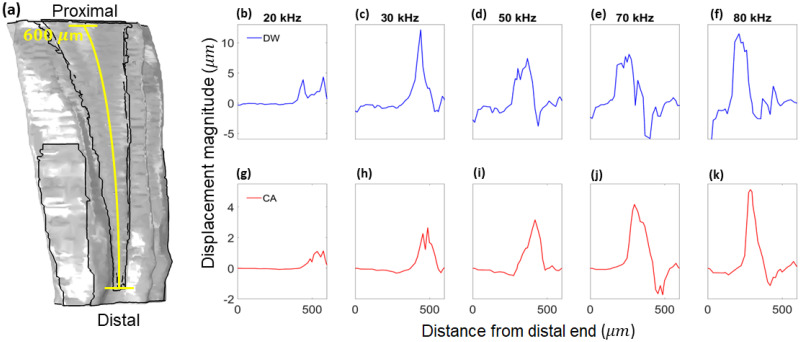
Displacement magnitudes. (a) The transect along which the displacement magnitudes were obtained. The displacement magnitude of the dorsal wall along the transect from (a), at (b) 20 kHz, (c) 30 kHz, (d) 50 kHz, (e) 70 kHz, and (f) 80 kHz. The displacement magnitude of the *crista acustica* along the transect from (a), at (g) 20 kHz, (h) 30 kHz, (i) 50 kHz, (j) 70 kHz, and (k) 80 kHz.

Further details on the DW and CA frequency dependent displacement is given in Fig 4, in the direction normal to the surfaces. The results were measured along the transect shown in Fig 4(a), with positive displacement in the direction of the outer normal and negative displacement in the direction of the inner normal. The transect is the centerline of both the DW and the CA surface. The displacement maxima were larger in magnitude along the DW compared to the CA.

Hence, the numerical results gave a qualitative match with the LDV and OCT recordings of tonotopic vibrations along the katydid hearing organ inferred from references ([11–13, 15, 16, 23, 32]).

### Driving force of tonotopy

**Fig 5:**
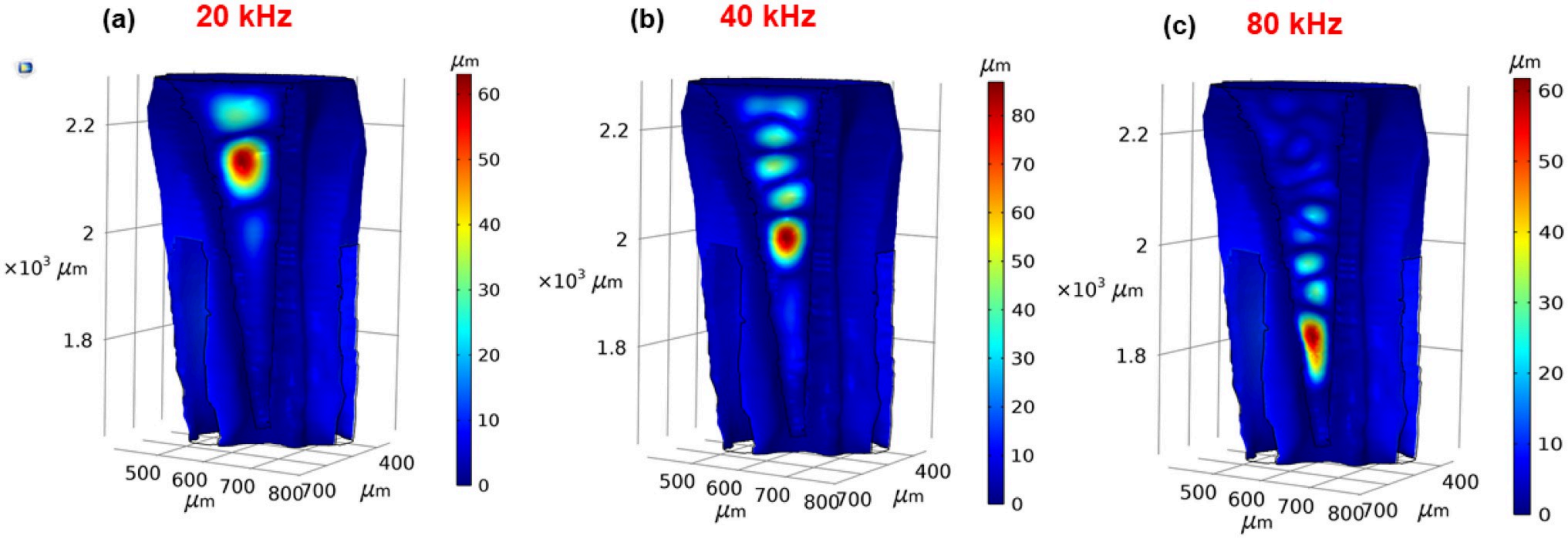
Dorsal wall tonotopy. The displacement magnitude of the dorsal wall at (a) 20 kHz, (b) 40 kHz, and (c) 80 kHz, after the removal of the *crista acustica*.

Following the qualitative match of the numerical results with experimental data (see [16] and references therein), the mathematical models were used to investigate the driving force of the tonotopic vibrations along the CA and the DW. To this end, first of all the CA was removed from the geometry. Hence the model was reduced to a fluid-structure interaction problem between the AV fluid and the AV wall. The results demonstrate frequency dependent displacements along the DW despite the removal of the CA (Fig 5).

**Fig 6:**
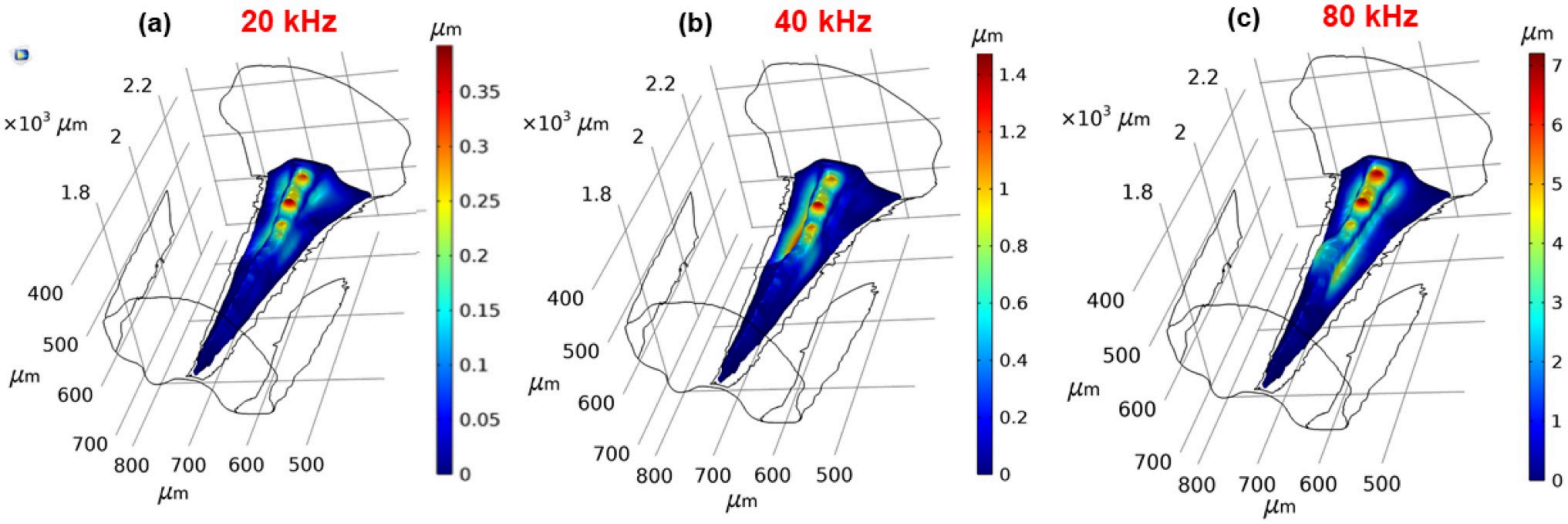
The solitary *crista acustica* influence on tonotopy. The displacement magnitude of the *crista acustica* at (a) 20 kHz, (b) 40 kHz, and (c) 80 kHz.

To investigate the individual effect of the CA on tonotopy, a new mathematical model was developed where it was assumed that the DW had the same properties as the remainder of the AV wall. Hence, the increasing thickness of the DW was removed and was assigned a uniform value of 15 *μ*m. The Young’s modulus of the DW was also taken to be the same as the remainder of the wall. The fluid-structure interaction between the AV fluid and the DW, and the solid-shell interaction between the CA and DW were still present. This allowed us to test for the independent effect of the CA on the frequency dependent displacement. In this scenario the maximum displacement was located at the proximal part of the CA for all frequencies considered (Fig 6).

**Fig 7:**
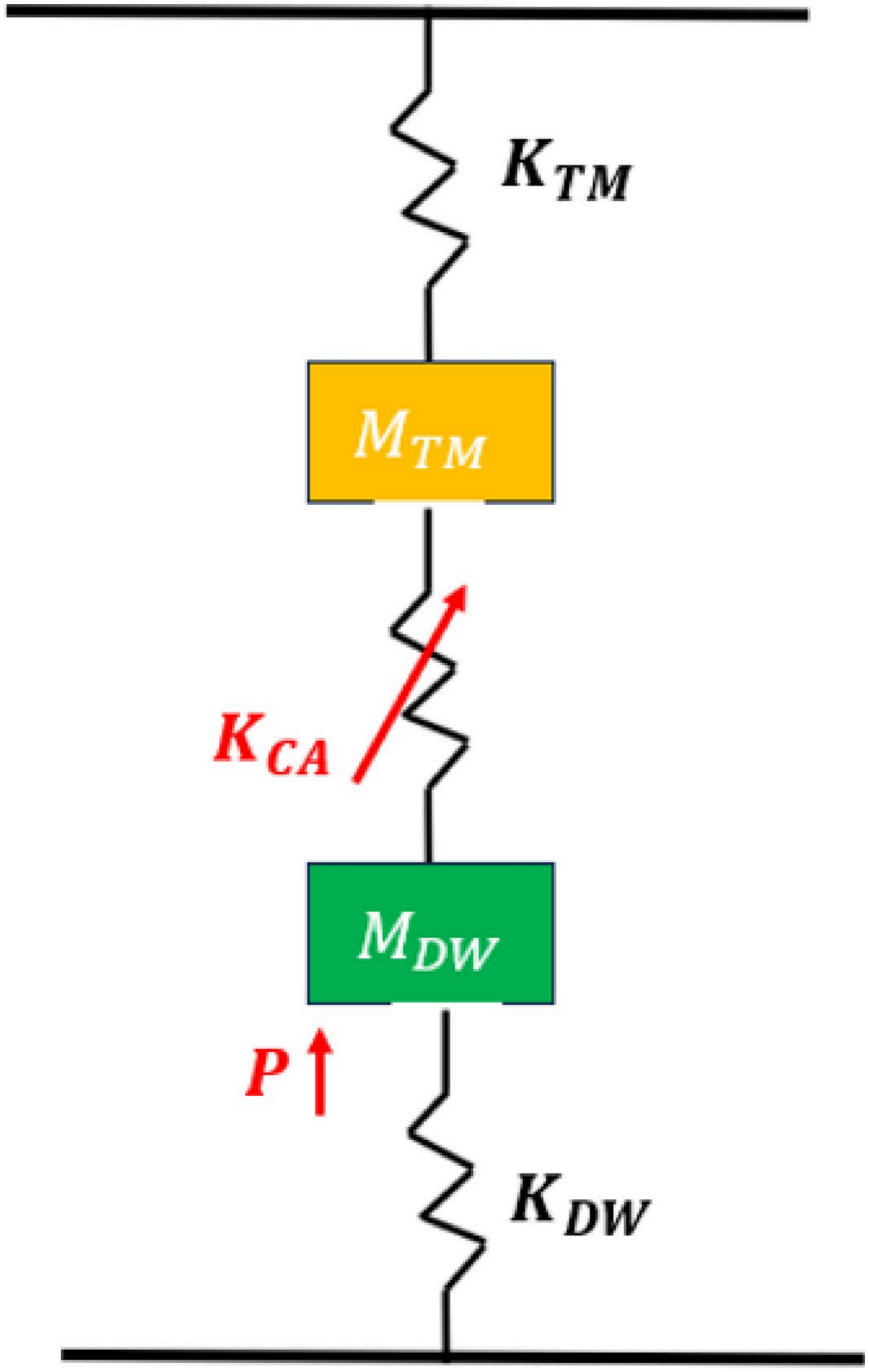
Mechanical network of a segment of the hearing organ. Abbreviations are as follows: *P*, pressure from the travelling wave; *M*_*DW*_, mass of dorsal wall; *K*_*DW*_, stiffness of dorsal wall; *K*_*CA*_, stiffness of *crista acustica* components; *M*_*TM*_, mass of tectorial membrane; *K*_*TM*_, stiffness of tectorial membrane. The source of displacements in the inner ear are shown in red. Vibrations of the dorsal wall are transmitted to the tectorial membrane via the *crista acustica* components dendrites and cap cells (red arrow).

Based on the numerical results from the two models considered above, the basic mechanical model of a segment of the katydid hearing organ is obtained (Fig 7). The DW displacements act as the driving force of tonotopy observed in the inner ear, which is activated by a pressure *P* representing the travelling wave vibrations. Since the removal of the mass and stiffness gradients along the DW led to the disappearance of tonotopic vibrations, their effect has been incorporated into the mechanical network, denoted *M*_*DW*_ and *K*_*DW*_, respectively. As a results of the displacements along the DW, the CA components are set in motion, whose stiffness is represented as *K*_*CA*_. In turn, this leads to displacements along the TM, which are the displacements we observe along the surface of the CA. The potential effect of the mass and stiffness gradients along the TM are also incorporated in the mechanical network as *M*_*TM*_ and *K*_*TM*_, respectively. The mechanical model does not contain damping effects as material damping was also not present in the numerical models.

### Travelling wave in the katydid inner ear

**Fig 8:**
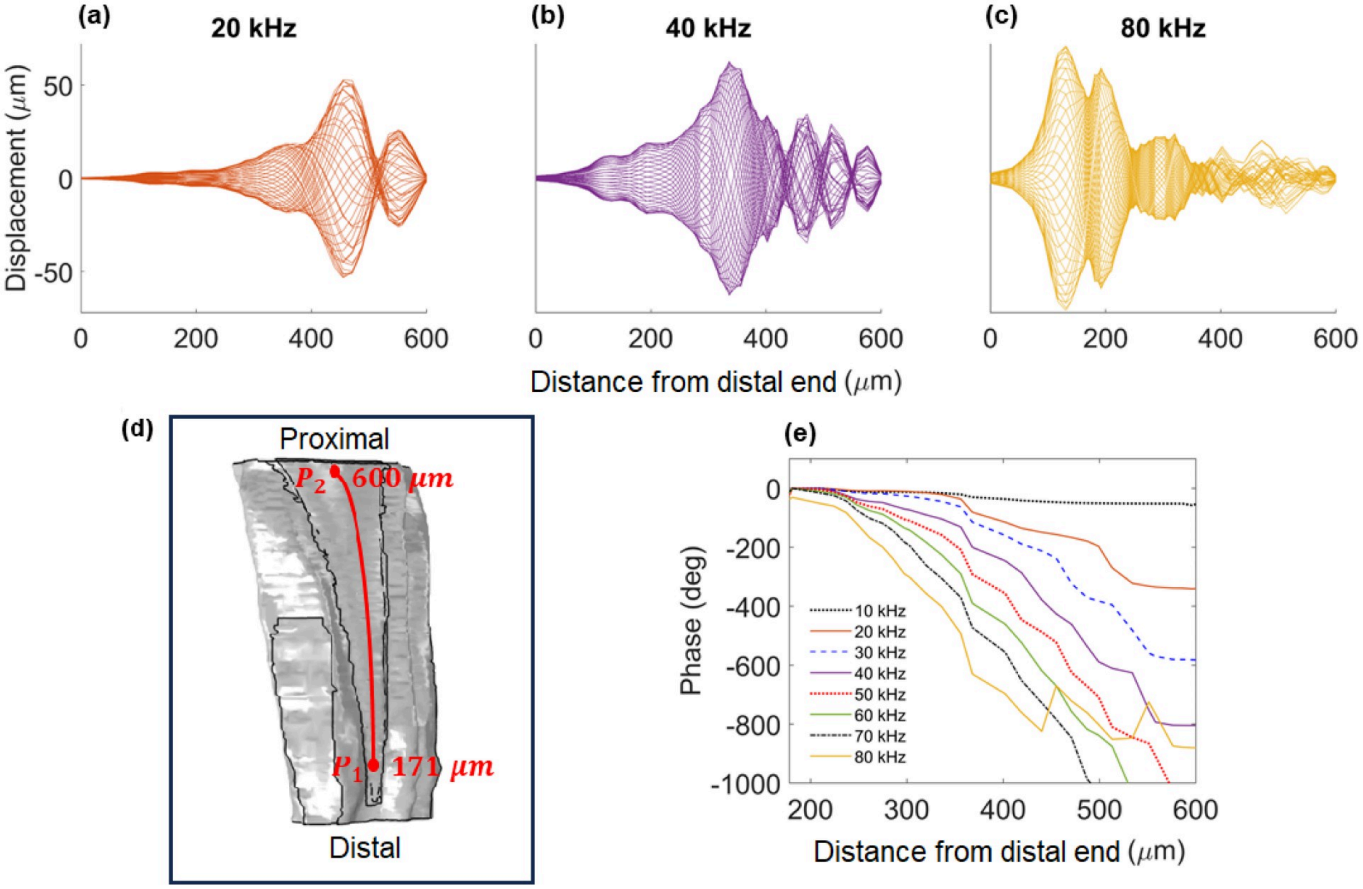
Travelling wave information. The time course of the dorsal wall vibrations at (a) 20 kHz, (b) 40 kHz, and (c) 80 kHz. (d) The transect used to measure the phase response between the points *P*_1_ and *P*_2_. (e) The phase response as the travelling wave moved along the transect in (d) in the frequency range 10–80 kHz.

To investigate whether the tonotopic vibrations observed in the katydid inner ear are guided by a travelling wave, we tested for an asymmetric envelope around the point of maximum displacement and an increasing phase lag with the increase of frequency. The numerical results were obtained using the time domain solutions at the frequencies 20 kHz, 40 kHz and 80 kHz. The displacement magnitude was measured along the transect shown in Fig 4(a), possessing a length of 600*μ*m. The magnitude of DW displacements showed an asymmetric envelope around the point of maximal displacement (Fig 8(a)–8(c)). Furthermore, the position of the maximum displacement moved from the distal end to the proximal end with the decrease of frequency. At 20 kHz, the wave was asymmetrical around 470 *μ*m from the distal end (Fig 8(a)), whereas for 40 kHz this was at 336 *μ*m (Fig 8(b)), and for 80 kHz this was around 130 *μ*m (Fig 8(c)).

Using the frequency domain results, we also considered the phase response along the transect between the points *P*_1_ and *P*_2_ (see Fig 8(d)), for the frequency range 10–80 kHz with a resolution of 10 kHz (Fig 8(e)). The results showed a significant increase in phase lag with the increase of frequency. At 10 kHz, the phase lag is 52°, which increases to 580° at 30 kHz, going up to 1000° for the higher end of the frequency range tested. As the waves traveled across the transect, the increase of frequency led to the phase lag appearing closer to the distal end.

Hence, the numerical results are in agreement with the response characteristics of a travelling wave.

## Discussion

### Validation of numerical simulations

The numerical results of our *in silico* model provided a qualitative match with the experimental tonotopy measurements along the katydid hearing organ in six previous studies ([12, 13, 15, 16, 23, 32]). The results were also in agreement with the OCT measurements by Vavakou et al. in [11], which exhibited frequency dependent displacements on both the DW and the CA (Fig 3).

The calling song frequency of *Mimetica* sp. is 12 kHz (Fig D(a) in S1 Text). The numerical results at 12 kHz did not show an increased response to the calling song frequency (Fig D(b)-(d) in S1 Text). Nevertheless, it is worth noting that the natural tuning of the tympana to the calling frequency was not incorporated into the mathematical model because of lack of experimental data on tympana impedance. The tympana impedance, however, would only have an effect on the displacement magnitude of the inner ear response to incoming vibrations and not on the location of the displacement response.

### Biomechanism behind tonotopy in the katydid ear

Following the validation of the numerical results, we used the mathematical models to go beyond experimental limitations, to understand the physiological basis of tonotopy in the katydid inner ear. In particular, we used the numerical models to determine if the tonotopic vibrations along the DW and CA existed independently of each other, by uncoupling these two structures. When the CA was removed from the geometry, a frequency dependent displacement similar to the one observed with the intact system still existed along the DW (Fig 5). This suggested that the mass and stiffness gradients of the DW resulting from its triangular design were sufficient for tonotopy. The removal of the CA, however, required the elastic modulus of the DW to be increased to 13 MPa, showing that the coupling of the dendrites with the DW had a stiffening effect on the structure. To test the independence of tonotopic vibrations along the CA from the DW, the DW thickness was set to a uniform value in the intact geometry. From the numerical results the location of the displacement maximum on the tectorial membrane did not move towards the distal end with the increase of frequency (Fig 6). Hence, the numerical results strongly suggested that the frequency dependent displacements of the hearing organ were driven by the DW, not the CA. The anatomical basis of tonotopy along the CA was examined previously by Hummel et al. [21], through detailed morphological measurements of the katydid *Mecopoda elongata* inner-ear and all of its components. The authors concluded that the height of the CA and the corresponding length of the sensory dendrites were best correlated to tonotopic frequency representation along the CA, due to the exponential decrease in dendrite height corresponding with the exponential distribution of the frequency dependent displacement maxima location. While our results do not support the conclusions drawn in Hummel et al., it is likely that the sensory dendrites and cap cells play a role in the spatial tuning of tonotopy. This can be observed, for instance, by a comparison of the displacement maxima location between when the system is intact and when the CA is removed (Fig E in S1 Text). This comparison shows some shifts in the location of the displacement maxima. Furthermore, these results show that the distance of the displacement maxima from the distal end decreases linearly with the increase of frequency when the CA is removed, whereas when the hearing organ is intact the distance of the displacement maximum from the distal end decreases more slowly with increasing frequency at higher frequencies. Hence, we show that the CA components contribute to the spatial tuning of the hearing organ.

### Travelling waves in the katydid inner ear

The numerical models were also used to show that the tonotopic vibrations of the katydid hearing organ were facilitated by the movement of a travelling wave along the CA, which carried the frequency information. The main conditions for the characterisation of travelling waves in the inner ear are the formation of an asymmetric envelope around the point of resonance and an increasing phase lag with the increase of frequency. The formation of the asymmetric envelope was observed from the time domain results, for input vibrations at 20, 40 and 80 kHz (see Fig 8(a)–8(c)). As the frequency domain results showed the vibrations along the DW to be the driving force of tonotopy in the katydid inner ear, time domain results were obtained using the geometry excluding the CA to reduce computational cost.

The phase lag of the wave along the DW was obtained in the frequency domain, along the transect specified at Fig 8(d). The results show the increase of the phase lag with frequency for the frequency range 10–80 kHz (Fig 8(e)). Hence, the results showed the main characteristics of a travelling wave were present along the DW. Even though travelling waves in the katydid inner ear had been observed experimentally ([12, 16, 22, 23]), to our knowledge, this is the first time a purely mechanical investigation of the process based on fluid dynamics and structural mechanics principles was carried out for the katydid hearing system. In summary, our numerical results provide theoretical evidence that the katydid inner ear can structurally support the formation of travelling waves for frequency analysis. The numerical results are also consistent with the *in vivo* measurements of travelling wave vibrations in the mammalian ear [3]. It is worth noting that, for the results the travelling wave does not completely dissipate after passing the point of resonance along the DW (Fig 8(a)–8(c)). This is mainly because of the absence of frequency dependent material damping in the mathematical model, due to lack of experimental data for the species *Mimetica* sp. Hence, the results are purely based on the mechanical structure of the DW and viscous damping due to fluid viscosity.

The magnitude of the displacements in the frequency domain (Fig 4) and the time domain compared well, where we used the results obtained without the CA for the frequency domain (Fig 5) to compare with the time domain data (Fig 8(a)–8(c)). In both cases, the displacement magnitudes scaled with the input vibration magnitude. For instance, reducing the input vibrations from a magnitude of 10*μ*m to 1*μ*m led to the scaling of the displacement magnitude by the same amount. This also held true for the results with the full geometry.

### Enhancement of mathematical models

Even though we developed and used comprehensive mathematical models to simulate the workings of the katydid inner ear, some simplifying assumptions still had to be made to reduce the computational cost or due to lack of experimental data. One such assumption was to exclude the air-filled acoustic trachea under the inner ear to reduce computational cost. The role of the acoustic trachea in providing input to the inner ear was investigated in detail in [20] with 3D numerical simulations on the idealised geometry of the katydid *Copiphora gorgonensis* inner ear. Here it was shown that while the inclusion of the trachea led to more pronounced tonotopic vibrations along the hearing organ, when only a tracheal input was considered (excluding the tympanal input), no tonotopy occurred. Hence, based on the results in [20], the inclusion of the tracheal input would not lead to a qualitative difference in the results, allowing us to exclude it from the model. We also assumed that there was no phase and amplitude difference between the two tympanic membranes, which can occur during the exterior input to tympana due to the direction of the sound stimulus [31]. Such a phase difference would certainly have an influence on spatial sound perception and affect the magnitude of the DW and CA displacement, and thus merits further investigation. Yet, it would not lead to a change in the mechanical network outlined in Fig 7. Another simplifying assumption relates to the AV fluid properties. The fluid measurements demonstrate that the AV fluid possesses the same density as water (Fig F(a) in S1 Text). Based on this, we made the simplifying assumption that the AV fluid also had the same viscosity as water, and thus incorporated the same properties as water at 20°C into the models. Preliminary, in house measurements of the AV fluid viscosity, however, have shown the fluid to be of varying viscosity, and generally higher in magnitude. Moreover, we assumed the AV fluid is Newtonian. While this assumption was based on the fluid showing Newtonian properties up to 25 Hz (Fig F(b) in S1 Text), the behaviour of the fluid at higher frequencies is still elusive. It is also worth noting that the fluid measurements were carried out using the species *C. gorgonensis* rather than *Mimetica* sp. This is due to *Mimetica* sp. having an opaque cuticle, rather than a transparent cuticle such as *C. gorgonensis*. Hence, the use of species with transparent cuticles allowed for the *in situ* measurements of fluid properties. Major differences are not expected in the AV fluid properties of different species, however, minor differences might be present. While such assumptions could potentially affect the displacement of the DW through small shifts in displacement maxima location and magnitude, it would not have a significant effect on the biopysical mechanism of the system.

A final simplifying assumption we made in the mathematical models was related to the material properties of the DW and the CA components, where we assumed them to be homogeneous and isotropic. Due to limited experimental data on the precise elastic modulus of the CA components, the incorporated material properties were determined through parametric sweeps in COMSOL [30], within the known bounds of insect cuticle [33] (see Section D in S1 Text). For the DW, in addition to parametric sweeps, our choice of elastic modulus was based on the recent measurements of Siamantouras et al. [34]. In this study, Atomic Force Microscopy was employed to measure the elastic modulus of a taenidial fibre along the acoustic trachea, which gave a wide distribution between 13.9 MPa and 26.5 GPa. The authors suggested that this was due to the taenidial fibre having a composite structure of resilin and chitin. The parametric sweeps we carried out in COMSOL gave the DW elastic modulus required for tonotopy as around 3 MPa, which was marginally outside of the range obtained in [34]. Hence, the numerical results suggested that the DW might have a resilin based (elastic) structure. Nevertheless, this difference can also be attributed to the general modelling assumptions adopted. It is also worth noting that the katydid species considered in [34] was *Copiphora gorgonensis*, which could have slight differences in its tracheal structure.

### Convergent evolution of the basic mechanical model of katydid and mammalian inner ears

Since the publication of von Bèkèsy’s Nobel prize winning work on cochlear mechanics [1], it has been predominantly accepted that sound transduction in the mammalian inner ear is driven by the displacement of the basilar membrane based on its mass and stiffness gradients [35]. While it is still acknowledged that the inner ear transduction is driven by the basilar membrane, further investigations into its workings have revealed that the other components of this coupled system also contribute to neural activity ([36–38]). Hence, a numerical model only accounting for the mechanical structure of the basilar membrane remains too simplistic to represent the workings of the inner ear [39]. A more complete mechanical network of the mammalian ear, including the effect of OHCs and the tectorial membrane, was suggested in [40], where the authors showed that the OHCs are essential for simulating the high sensitivity and sharp tuning characteristic of the mammalian cochlea. While for the mammalian ear it was initially believed that tonotopy was exclusively led by the basilar membrane, with the katydid inner ear it was conjectured that the inner ear components such as dendrites and cap cells drove the mechanism [21]. Our numerical investigation into the katydid inner ear, however, revealed an analogous biomechanism behind the tonotopic vibrations as the mammalian cochlea. The mechanical network arising from the numerical results (Fig 7) showed the DW to have an equivalent role to the mammalian basilar membrane. Based on our results, we propose that the DW acts as the dominant component in the development of tonotopic vibrations along the hearing organ, with the CA components playing a comparably minor role by contributing to the tuning of tonotopy. The precise contributions of the CA components to the katydid inner ear biomechanism, as well as the mechanotransduction leading to neural activity (such as the stretching of dendrites or the tilting of scolopale cells [11, 41]) are beyond the scope of this study, and would require the development of an electromechanical model for capturing the extra purely ionic filtering step within the cilium during the transduction of the displacements into neural potentials. For a more complete understanding of the katydid inner ear biophysical mechanism, such an electromechanical model would certainly merit future investigation. In conclusion, we show that both mammals and katydids have resolved the problem of frequency analysis in analogous but evolutionarily independent ways. Despite the katydid and mammals employing very different molecular building blocks (cuticle, trachea, microtubule-based cilia vs bone, ear canal and actin-based cilia) both animals solved the need for frequency discrimination using an analogous biophysical mechanism. Impressively, katydids are able to perform this sophisticated process using a hearing organ less than 1 mm in length. Hence, our results show that katydid hearing is not compromised biophysically by their small size, and there is a lot we can learn from their ingenious methods of overcoming such limitations.

## Conclusion

In this study, we took an interdisciplinary approach to investigate the katydid inner ear mechanism behind the frequency dependent displacements within their inner ear. The investigation was carried out using robust, 3D numerical simulations, which incorporated: *(i)* the precise geometry of the inner ear and its components, obtained by synchrotron-based *μ*–CT scanning and 3D reconstruction, and *(ii)* AV fluid properties obtained by a volumetric method and *in situ* multiple particle tracking microrheology.

The numerical results showed that tonotopy in the katydid inner ear was driven by the vibrations of the DW the CA is placed on. This suggests that the DW has a similar function to the basilar membrane in the mammalian ear. Hence, the results showed a prominent analogy between the mechanical networks of the mammalian and katydid inner ear workings. This introduces the possibility of adopting the comparably simpler yet analogous katydid auditory system as a model hearing system for the continued investigation of the complex mammalian auditory mechanism [42]. Through their similarities, as well as differences, such a comparison will undoubtedly improve our understanding of both hearing systems.

## Supporting information

S1 Text Supplementary text, tables and figures. (PDF)
