# Supplementary material for: Mechanical network equivalence between the katydid and mammalian inner ears
Source: PLoS Comput Biol. 2024 Dec 13;20(12):e1012641. doi: 10.1371/journal.pcbi.1012641 (PMC11676532; doi:10.1371/journal.pcbi.1012641)
Supplement: S1 Text — (PDF) [file pcbi.1012641.s001.pdf]

Mechanical network equivalence between the katydid and mammalian inner ears
Supplementary Materials

Emine Celiker, Charlie Woodrow, Òscar Guadayol,
Leonidas-Romanos Davranoglou, Christian M. Schlepütz, Beth Mortimer,
Graham K. Taylor, Stuart Humphries, Fernando Montealegre-Z

A Tomographic data processing measurements

The parameters used in collecting the synchrotron-based micro-computed tomography data are listed in Figure A.

```
--------------------Beamline Settings-------------------------
Ring current [mA]            : 401.588
Beam energy [keV]            : 16.000
Monostripe                   : Ru/C
FE-Filter                    : Filter 50%
OP-Filter 1                  : 100um Al
OP-Filter 2                  : No Filter
OP-Filter 3                  : No Filter
--------------------Detector Settings-------------------------
Camera                       : PCO.Edge 5.5
Microscope                   : Opt.Peter 4x op
Magnification                : 4.0
Scintillator                 : LuAg:Ce 150um (C150f)
Exposure time [ms]           : 30.0
Delay time [ms]              : 0.0
Millisecond shutter [ms]     : not used
X-ROI                        : 1 - 2560
Y-ROI                        : 1 - 2160
Actual pixel size [um]       : 1.625
------------------------Scan Settings-------------------------
Sample folder                : /sls/X02DA/data/e19549/Data10/disk2/A_mim_sp_m_/
File Prefix                  : A_mim_sp_m_
Number of projections        : 2000
Number of darks              : 30
Number of flats              : 150
Number of inter-flats        : 0
Flat frequency               : 0
Rot Y min position  [deg]    : 0.0
Rot Y max position  [deg]    : 180.0
Rotation axis position       : Standard
Angular step [deg]           : 0.090
Double first/last projection : True
Sample In   [um]             :     0
Sample Out  [um]             : 10000
-----------------------Sample coordinates---------------------
X-coordinate                 : 526.50
Y-coordinate                 : 3600.00
Z-coordinate                 : 3660.00
XX-coordinate                : -805.82
ZZ-coordinate                : 2567.97
ROTX-rotation                : -2120.80
----------------------Microscope coordinates--------------------
X-coordinate                 : -37.32
Y-coordinate                 : -158.46
Z-coordinate                 : 218.40
```

Figure A: Parameters used in collecting the tomographic data.

B The Mathematical Model

The inner ear (auditory vesicle, AV) of the bush-cricket *Mimetica sp.* was modelled as the union of three different types of domains:

1. The *fluid domain*, which was the liquid inside the AV;
2. The *solid domain*, comprised of the cap cells and dendrites of the hearing organ *crista acustica* (CA);
3. The *shell domain*, which contained the walls of the AV including the dorsal wall (DW), the tectorial membrane covering the CA components, and the tympanal plates.

For a frequency domain solution, the following system of equations was employed in the fluid domain:

$$0 = i\omega\rho + \nabla\cdot(\rho_0\mathbf{u}_f), \tag{1}$$

$$i\omega\rho_0\mathbf{u}_f = \nabla\cdot\left[-pI + \mu(\nabla\mathbf{u}_f + (\nabla\mathbf{u}_f)^T) - (\frac{2\mu}{3} - \mu_B)(\nabla\mathbf{u}_f)I\right], \tag{2}$$

$$\nabla\cdot(k\nabla T) = i\omega\rho_0 C_p T + \mathbf{u}\cdot\nabla T_0 - i\omega\alpha_p T_0 p + \mathbf{u}\cdot\nabla p_0 \tag{3}$$

$$\rho = \rho_0(\beta_T p - \alpha_p T), \tag{4}$$

where equations (1)-(4) are the continuity equation, the linearized Navier-Stokes equations, the energy equation and the linearized equation of state, respectively. The equation parameters are $i = \sqrt{-1}$; $\omega =$ angular frequency; $\rho =$ density, obtained from the linearized equation of state; $\rho_0(p_0, T_0) =$ equilibrium density; $\mu =$ dynamic viscosity; $\mu_B =$ bulk viscosity; $k =$thermal conductivity; $C_p =$ heat capacity at constant pressure; $T_0 =$ equilibrium temperature; $p_0 =$ equilibrium pressure; $I =$ identity matrix; $\alpha_p =$ coefficient of thermal expansion and $\beta_T=$ the isothermal compressibility. Fluid parameter values are based on the properties of water at 20 °C. The dependent variables are $p =$ pressure, $\mathbf{u}_f =$ velocity and $T =$ temperature.

The elastic Helmholtz equation,

$$\mathbf{0} = -\rho_s\omega^2\mathbf{U}_s - \nabla\cdot\sigma(\mathbf{U}_s), \tag{5}$$

was applied on the solid domain, where $\rho_s =$ density, $\sigma_{ij} = \frac{E}{1+\nu}\epsilon_{ij} + \frac{E\nu}{(1+\nu)(1-2\nu)}\epsilon_{kk}\delta_{ij},\ i,j = 1,2,3,$ $E =$ Young's modulus; $\nu =$ Poisson's ratio; $\epsilon_{ij} = \frac{1}{2}\left(\frac{\partial\mathbf{U}_{si}}{\partial x_j} + \frac{\partial\mathbf{U}_{sj}}{\partial x_i}\right)$ the strain tensor and δ_{ij} the Kronecker-delta function. The dependent variable $\mathbf{U}_s$ represents the displacement field of the solid domain. The parameter values incorporated into the model are given in Table A.

Table A: The parameter values incorporated to the equations representing the workings in the solid domain.

Parameter	Value
ρ_s (Cap cells)	1300 kg/m^3
ρ_s (Dendrites)	1200 kg/m^3
E (Cap cells)	10 MPa
E (Dendrites)	20 MPa
ν	0.3

The cap cells and dendrites, whose displacements are represented by equation (5) for each component separately, are in reality attached. To model the connection between these components realistically, a continuity condition is introduced on their shared boundaries.

For obtaining the displacements of the shell domain, the shell interface of COMSOL Multiphysics, v. 5.6, was applied [1]. The shell was assumed to be comprised of a linear elastic material so that the linear elastic equation node of COMSOL Multiphysics was assigned for the solution at its midplane. The shell was assumed to be isotropic, and four separate linear elastic material nodes were applied for the DW, tectorial membrane, pressure release facilitator and the remainder of the AV walls. These

were further coupled with the thickness and offset nodes, so that each equation incorporated individual material properties and thicknesses. The Poisson's ratio and density were taken as 0.3 and 1300 kg/m^3, respectively, for all the shell boundaries. For a unique solution, and to ensure continuity between the different boundaries, fixed constraints were assigned to the edges of the shell domain, so that equations (6), (7)

$$\mathbf{U}_{sh} = \mathbf{0}, \tag{6}$$

$$\mathbf{ar} = \mathbf{0}, \tag{7}$$

were satisfied on the shell edges, where $\mathbf{U}_{sh}$ is the shell displacement field and **ar** is the displacement of shell normals. The system was excited by the prescribed acceleration conditions given by equations (8) and (9), which were applied at the tympanal plates,

$$-\omega^2 \mathbf{U}_{sh,x} = -10\omega^2\ \mu m, \tag{8}$$

$$-\omega^2 \mathbf{U}_{sh,y} = -10\omega^2\ \mu m, \tag{9}$$

where ω is the angular frequency as defined above.

The fluid and shell systems were coupled at their intersections by the equations (10) and (11),

$$\mathbf{u}_f = i\omega \mathbf{U}_{sh}, \tag{10}$$

$$T = 0, \tag{11}$$

where the complex number $i = \sqrt{-1}$.

Finally, the solid and shell boundaries were coupled at parallel boundaries through the solid-thin structure connection node of COMSOL Multiphysics, v. 5.6.

The mathematical model for the time domain solution was the time dependent equivalent of the system detailed above.

C Numerical Simulations

The numerical solution to the system of equations given in Section B was obtained using the simulation toolbox COMSOL Multiphysics (v. 5.6), [1]. In the frequency domain, the finite element method was employed for the solution to the variational form of the used system of equations. A visual representation of the constructed finite element mesh is given in Figure B. Linear and quadratic Lagrange elements were used for the solution in the fluid domain and solid/ shell formulations, respectively, [2]. The radii of the tetrahedral meshes forming the finite element mesh in the domain were 1.02 - 23.7 μm (see Figures Ba, Bc). By the formula

$$\delta_\nu = \sqrt{\frac{\mu}{\pi f \rho_0}},$$

where μ =dynamic viscosity, ρ_0 = density and δ_ν = viscous layer thickness, at 20 °C the viscous layer thickness of water ranges between 1.99 - 5.65 μm for the frequency range considered. Hence the used finite element mesh allowed for capturing the viscous layer effects. Moreover, the employed mesh ensured that there were at least 10 finite elements per wavelength for the maximum frequency of 80 kHz considered. For the solution on the parts of the boundary represented with the shell formulation, mixed interpolation of tensorial components (MITC) shell elements were used [3].

For the time domain calculations, at each time step the solution was obtained using the finite element method as described above. The time-dependent solver used was the generalized alpha method, available in COMSOL Multiphysics. A small time step of $\Delta t = 1/(1024$ kHz$)$=0.977 μs was used to ensure numerical stability. In total 4096 time steps were taken. As each simulation started from rest, the results were recorded after 2048 time steps, so that the initial oscillatory transient response was smoothed out.

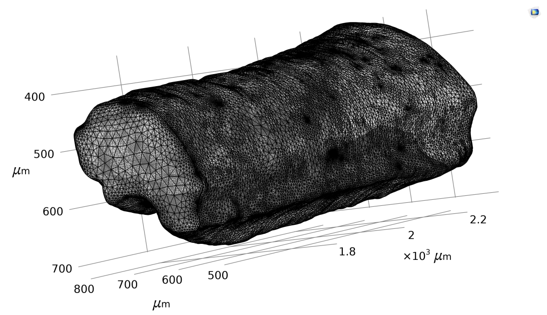

(a) Finite-element mesh formed in the auditory vesicle.

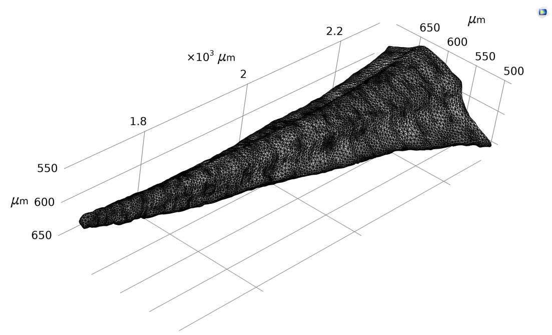

(b) Triangular elements along the tectorial membrane.

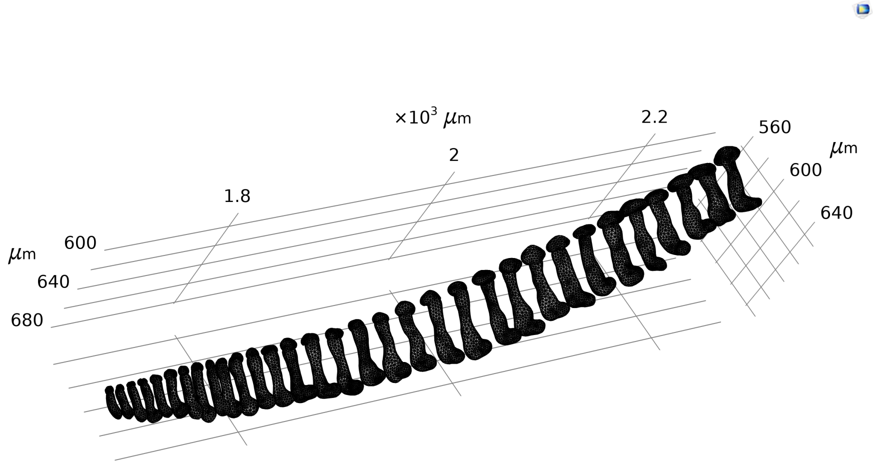

(c) Finite-element mesh on the cap cells and the dendrites.

Figure B: The finite element mesh constructed for obtaining the approximate solution in (a) the fluid domain, (b) a boundary of the shell domain and (c) the solid domain.

D Parameter material properties

The Young's modulus (E) of the inner-ear parameters were determined using parametric sweeps of the values given in Table B and combinations there of. The values in Table B were selected based on known bounds of insect cuticle [4], [5]. The values used in the final models were dependent on the quality of the tonotopical pattern of vibration obtained in the intact inner ear geometry.

Table B: The Young's modulus and thickness of components used in the mathematical model.

**Parameter**	**Young's Modulus** (GPa)
Dorsal wall	0.001, 0.003, 0.005, 0.1, 0.2, 0.5
Acoustic vesicle wall	2, 10, 15, 20
Cap cell	0.001, 0.01, 0.05, 0.1
Dendrite	0.001, 0.01, 0.02, 0.05, 0.1
Tectorial membrane	0.001, 0.01, 0.05, 0.1
Tympanal plate	0.5, 1, 2, 5
Pressure release facilitator	0.01, 0.025, 0.05, 0.1, 1, 2, 5

E Further Numerical Results

Figure C demonstrates the displacement of the small components of the *crista acustica*, cap cells and dendrites, at 20, 40 and 80 kHz. The increase in frequency leads to an increase in the total displacement magnitudes of the components.

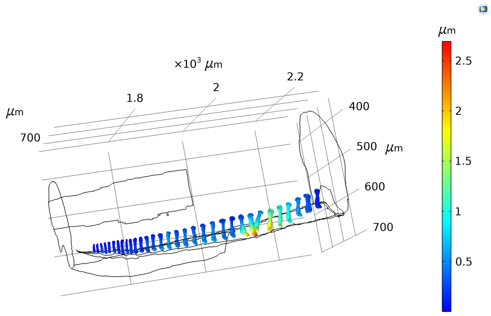

(a) The total displacement magnitudes of cap cells and dendrites at 20 kHz.

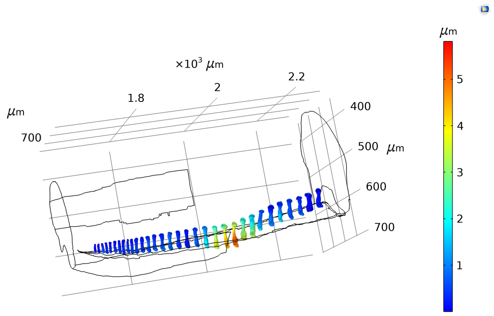

(b) The total displacement magnitudes of cap cells and dendrites at 40 kHz.

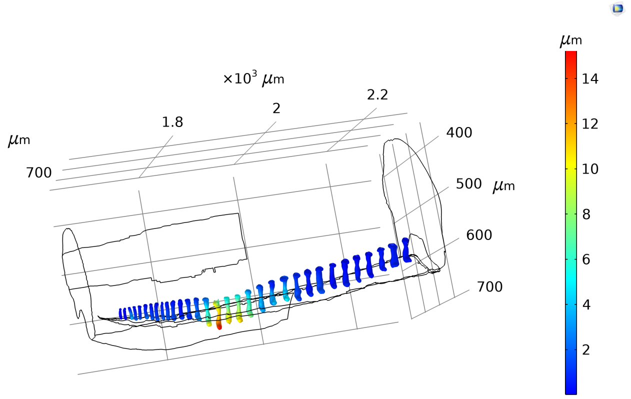

(c) The total displacement magnitudes of cap cells and dendrites at 80 kHz.

Figure C: The displacements of the *crista acustica* components at (a) 20 kHz, (b) 40 kHz, and (c) 80 kHz.

F Calling Song Frequency

The bush-cricket species *Mimetica sp.* has a calling song frequency of 12 kHz. Figure D demonstrates the displacement of the *Mimetica sp.* inner ear components at 12 kHz.

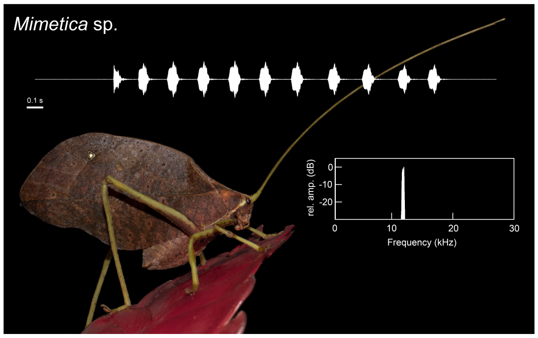

(a) The spectral decomposition of *Mimetica* sp. calling song. The spectrum is plotted from the entire waveform.

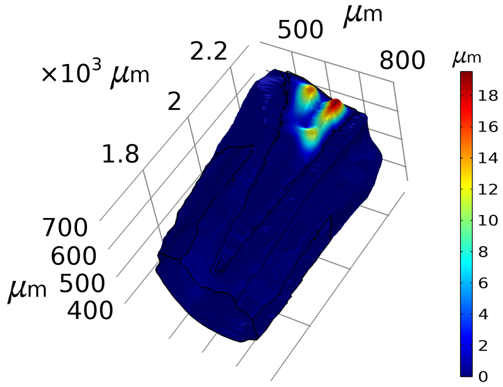

(b) The total displacement magnitude of the dorsal wall at 12 kHz.

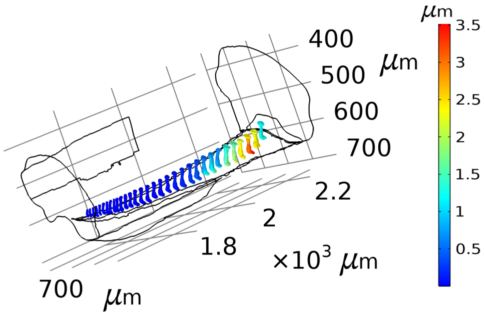

(c) The total displacement magnitudes of cap cells and dendrites at 12 kHz.

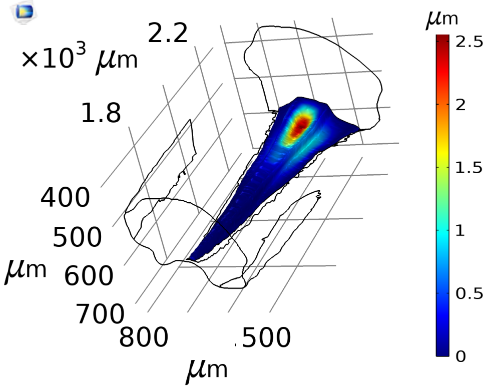

(d) The total displacement magnitude of the tectorial membrane at 12 kHz.

Figure D: Numerical simulation of the inner ear response to *Mimetica sp.* calling song. (a) *Mimetica* sp. and the spectral decomposition of its calling song. The displacement magnitudes of the (b) dorsal wall, (c) cap cells and dendrites, and (d) tectorial membrane at the calling song frequency of 12 kHz.

G Displacement maxima location in the katydid inner ear

Figure E demonstrates the location of the displacement maximum in the extended frequency range 10-100 kHz. The two cases compared are the displacement maxima location along the hearing organ, *crista acustica*, and along the dorsal wall after the *crista acustica* has been removed. The results show that when the hearing organ is intact, there is a slower decrease in the distance from the distal end at the higher frequencies considered.

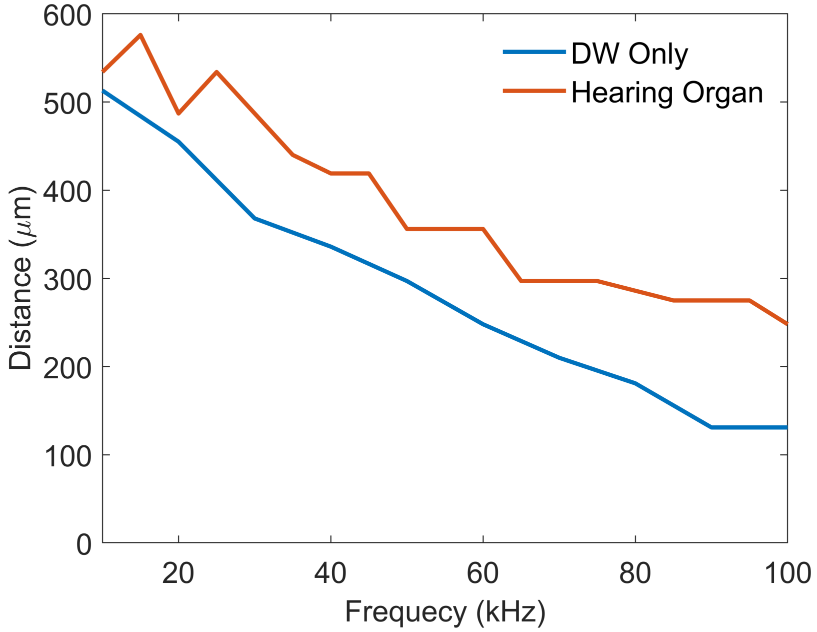

Figure E: The maximum displacement location of the *Mimetica* sp. hearing organ, and the dorsal wall after the hearing organ has been removed.

H Results of Auditory Vesicle fluid properties measurements

Figure Fa demonstrates that the density of ultrapure water and the AV fluid are comparable due to having the same weight to volume ratios. Figure Fb shows the rheological behaviour of the AV fluid.

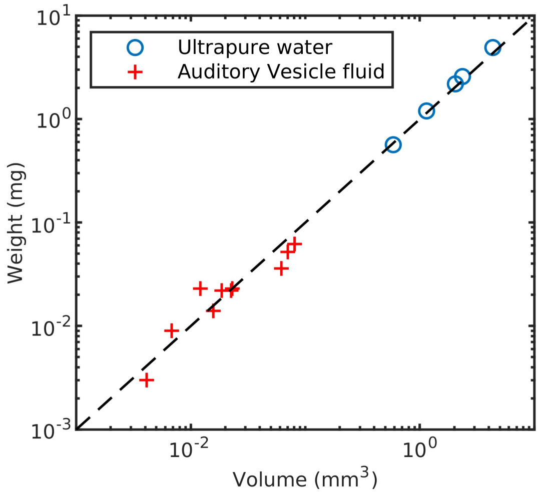

(a) Volume vs Weight of AV fluid and ultrapure water.

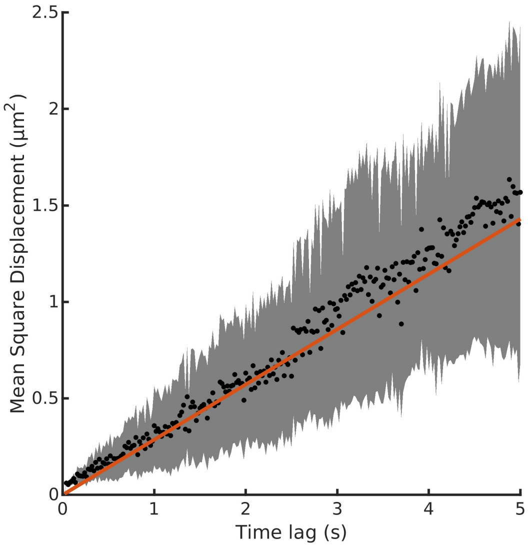

(b) Rheological behaviour of AV fluid.

Figure F: The Auditory Vesicle fluid properties. (a) Volume and weight measurements used to calculate AV fluid density, and the ultrapure density for comparison. (b) The Mean Squared Displacement (MSD) of a population of polyethylene microspheres of 1.02 μm in diameter suspended in the AV fluid. Data points show the ensemble MSD estimates per time lag τ, and grey areas represent the standard error of the estimates. The red line shows the least squares fit to $\mathrm{MSD}(\tau) = 4\mathrm{D}\tau$, where D is the empirical diffusion coefficient of the microspheres in the AV fluid.

References

[1] Comsol Multiphysics v. 5.6, www.comsol.com, Comsol AB, Stockholm, Sweden.

[2] Brenner, S. C., Scott, L. R., & Scott, L. R. (2008). *The mathematical theory of finite element methods (Vol. 3)*. New York: Springer.

[3] Bathe, K. J., Iosilevich, A., & Chapelle, D. (2000). An evaluation of the MITC shell elements. *Computers & Structures*, **75**(1), 1-30.

[4] Vincent, J. F., & Wegst, U. G. (2004). Design and mechanical properties of insect cuticle. *Arthropod structure & development*, **33**(3), 187-199.

[5] Siamantouras, E., Woodrow, C., Celiker, E., Cullen, D. A., Hills, C. E., Squires, P. E., & Montealegre-Z, F. (2022). Quantification of bush-cricket acoustic trachea mechanics using Atomic Force Microscopy nanoindentation. *Acta Biomaterialia*, **153**, 399-410.
